# Supplementary material for: SARS-CoV-2 Spike and Neutralizing Antibody Kinetics 90 Days after Three Doses of BNT162b2 mRNA COVID-19 Vaccine in Singapore
Source: Vaccines (Basel). 2022 Feb 18;10(2):331. doi: 10.3390/vaccines10020331 (PMC8879250; doi:10.3390/vaccines10020331)
Supplement: Supplementary file 1 [file vaccines-10-00331-s001.zip › vaccines-1595419-supplementary.pdf]

Supplementary Table S1: STARD checklist

Section & Topic	No	Item	Reported on page #
**TITLE OR ABSTRACT**			
	**1**	Identification as a study of diagnostic accuracy using at least one measure of accuracy (such as sensitivity, specificity, predictive values, or AUC)	**2**
**ABSTRACT**			
	**2**	Structured summary of study design, methods, results, and conclusions (for specific guidance, see STARD for Abstracts)	**2**
**INTRODUCTION**			
	**3**	Scientific and clinical background, including the intended use and clinical role of the index test	**4**
	**4**	Study objectives and hypotheses	**4**
**METHODS**			
Study design	**5**	Whether data collection was planned before the index test and reference standard<br>were performed (prospective study) or after (retrospective study)	**5**
Participants	**6**	Eligibility criteria	**5**
	**7**	On what basis potentially eligible participants were identified (such as symptoms, results from previous tests, inclusion in registry)	**5**
	**8**	Where and when potentially eligible participants were identified (setting, location and dates)	**5**
	**9**	Whether participants formed a consecutive, random or convenience series	**5**
Test methods	**10a**	Index test, in sufficient detail to allow replication	**5**
	**10b**	Reference standard, in sufficient detail to allow replication	**5**
	**11**	Rationale for choosing the reference standard (if alternatives exist)	**5**
	**12a**	Definition of and rationale for test positivity cut-offs or result categories of the index test, distinguishing pre-specified from exploratory	**5**
	**12b**	Definition of and rationale for test positivity cut-offs or result categories of the reference standard, distinguishing pre-specified from exploratory	**5**
	**13a**	Whether clinical information and reference standard results were available<br>to the performers/readers of the index test	**5**
	**13b**	Whether clinical information and index test results were available to the assessors of the reference standard	**5**
Analysis	**14**	Methods for estimating or comparing measures of diagnostic accuracy	**6**
	**15**	How indeterminate index test or reference standard results were handled	**6**
	**16**	How missing data on the index test and reference standard were handled	**6**
	**17**	Any analyses of variability in diagnostic accuracy, distinguishing pre-specified from exploratory	**6**
	**18**	Intended sample size and how it was determined	**Not applicable**
**RESULTS**			

Participants	**19**	Flow of participants, using a diagram	**Not applicable**
	**20**	Baseline demographic and clinical characteristics of participants	**5**
	**21a**	Distribution of severity of disease in those with the target condition	**Not applicable**
	**21b**	Distribution of alternative diagnoses in those without the target condition	**Not applicable**
	**22**	Time interval and any clinical interventions between index test and reference standard	**Not applicable**
Test results	**23**	Cross tabulation of the index test results (or their distribution) by the results of the reference standard	**7**
	**24**	Estimates of diagnostic accuracy and their precision (such as 95% confidence intervals)	**Not applicable**
	**25**	Any adverse events from performing the index test or the reference standard	**Not applicable**
**DISCUSSION**			
	**26**	Study limitations, including sources of potential bias, statistical uncertainty, and generalisability	**11**
	**27**	Implications for practice, including the intended use and clinical role of the index test	**11**
**OTHER INFORMATION**			
	**28**	Registration number and name of registry	**6**
	**29**	Where the full study protocol can be accessed	**Not applicable**
	**30**	Sources of funding and other support; role of funders	**Not applicable**

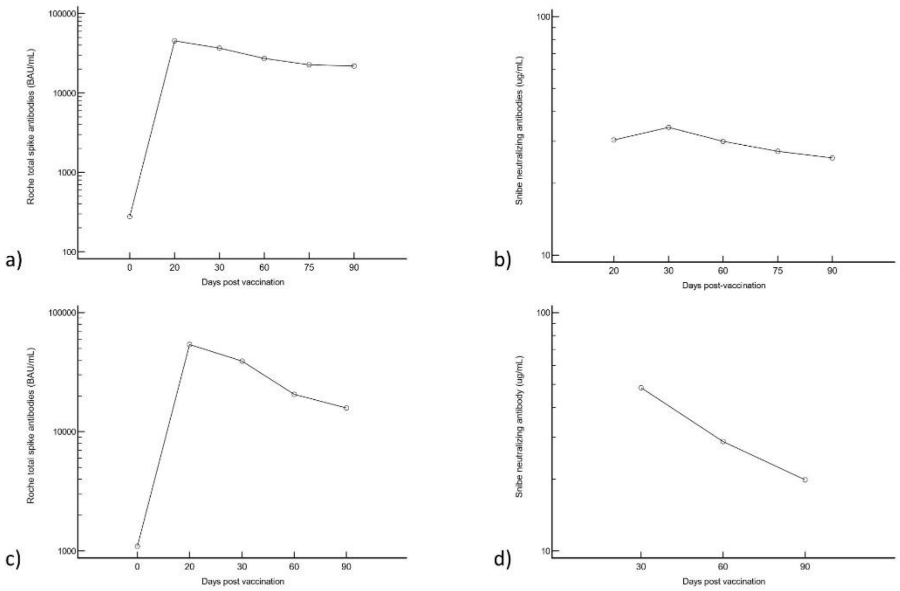

Supplementary Figure S1: 2 subjects had antibody testing at all time points on the Roche total spike assay from pre-booster to 90 days booster vaccination, with Snibe neutralizing antibodies tested at almost all time points. a) Roche and b) Snibe antibody kinetics for case 1, and c) Roche and d) Snibe antibody kinetics for case 2 are displayed on a semi-logarithmic scale.

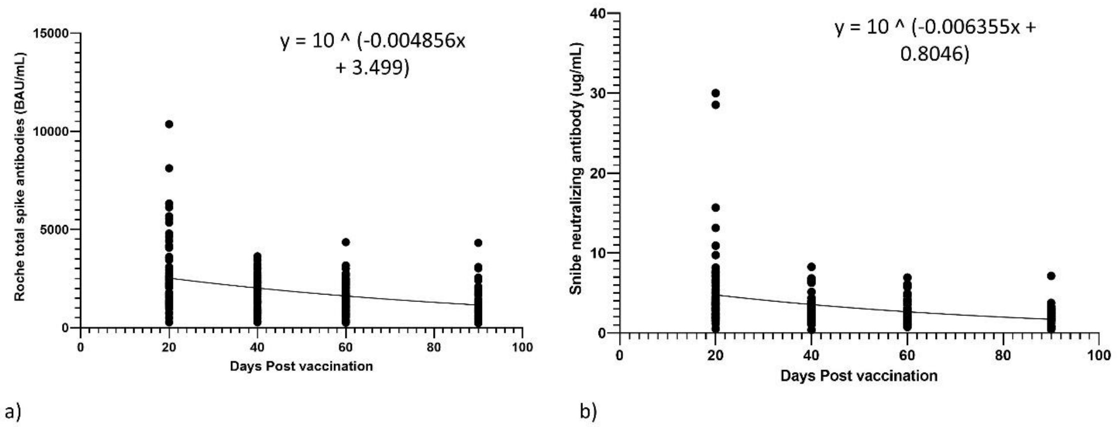

Supplementary Figure S2: Non linear regression of a) Roche total spike and b) Snibe neutralizing antibodies from their peak at 20 days post-second dose vaccination, based on our prior data set [12].
